# IKKγ/NEMO Localization into Multivesicular Bodies

**DOI:** 10.3390/ijms23126778

**Published:** 2022-06-17

**Authors:** Lisa-Marie Wackernagel, Mohsen Abdi Sarabi, Sönke Weinert, Werner Zuschratter, Karin Richter, Klaus Dieter Fischer, Ruediger C. Braun-Dullaeus, Senad Medunjanin

**Affiliations:** 1 Department of Internal Medicine, Division of Cardiology and Angiology, Otto-von-Guericke University, 39120 Magdeburg, Germany; lisa.schleithoff@gmx.de (L.-M.W.); mohsen.abdi@med.ovgu.de (M.A.S.); soenke.weinert@med.ovgu.de (S.W.); r.braun-dullaeus@med.ovgu.de (R.C.B.-D.); 2 Leibniz Institute for Neurobiology, 39120 Magdeburg, Germany; werner.zuschratter@lin-magdeburg.de; 3 Institute of Biochemistry and Cell Biology, Otto-von-Guericke University, 39120 Magdeburg, Germany; karin.richter@med.ovgu.de (K.R.); klaus.fischer@med.ovgu.de (K.D.F.)

**Keywords:** NF-κB pathway, NF-κB essential modifier (NEMO/IKKγ), glycogen synthase kinase-3 (GSK-3)

## Abstract

The NF-κB pathway is central pathway for inflammatory and immune responses, and IKKγ/NEMO is essential for NF-κB activation. In a previous report, we identified the role of glycogen synthase kinase-3β (GSK-3β) in NF-κB activation by regulating IKKγ/NEMO. Here, we show that NEMO phosphorylation by GSK-3β leads to NEMO localization into multivesicular bodies (MVBs). Using the endosome marker Rab5, we observed localization into endosomes. Using siRNA, we identified the AAA-ATPase Vps4A, which is involved in recycling the ESCRT machinery by facilitating its dissociation from endosomal membranes, which is necessary for NEMO stability and NF-κB activation. Co-immunoprecipitation studies of NEMO and mutated NEMO demonstrated its direct interaction with Vps4A, which requires NEMO phosphorylation. The transfection of cells by a mutated and constitutively active form of Vps4A, Vps4A-E233Q, resulted in the formation of large vacuoles and strong augmentation in NEMO expression compared to GFP-Vps4-WT. In addition, the overexpression of the mutated form of Vps4A led to increased NF-κB activation. The treatment of cells with the pharmacologic V-ATPase inhibitor bafilomycin A led to a dramatic downregulation of NEMO and, in this way, inhibited NF-κB signal transduction. These results reveal an unexpected role for GSK-3β and V-ATPase in NF-κB signaling activation.

## 1. Introduction

A central player of the innate immune system is the transcription factor Nuclear Factor κB (NF-κB) [1]. Two signaling pathways lead to its activation: the canonical and the non-canonical [2]. The canonical is designated by the IKK complex, consisting of IKKα, IKKβ, and the regulatory subunit IKKγ/NF-κB essential modulator (NEMO) [3]. While IKKα and IKKβ are catalytic subunits, the IKKγ/NEMO dimer shows no catalytic activity [3,4]; nevertheless, it is strictly required for canonical NF-κB activation [5]. The transcription factors of the NF-κB family play a pivotal role in the cellular response to DNA-damage such like ionizing radiation [6,7,8]. This non-canonical pathway is also regulated by NEMO, which shuttles through the nucleus by undergoing different post-translational modifications such as phosphorylation, SUMOylation and ubiquitylation [9,10] before binding to the IKK complex, resulting in subsequent NF-κB activation.

Given its ubiquitous expression throughout various cell types and its role in multiple cellular functions, NEMO has been implicated in various inflammatory diseases. Furthermore, mutations of the NEMO gene, are the cause of Incontinentia Pigmenti (IP) and Anhidrotic Ectodermal Dysplasia with Immunodeficiency (AED-ID) [11,12]. The mutation is mostly seen in female patients due reduced NF-κB activity, which leads to the mutation in males in utero, which has fatal consequences [11]. In mice, the ablation of NEMO results in a lack of detectable NF-κB DNA-binding activity and an embryonic lethal phenotype because of severe liver damage due to massive apoptosis [13].

Glycogen synthase kinase 3 (GSK3) is an evolutionarily conserved enzyme identified as a negative regulator of glycogen synthesis [14]. Since its initial discovery, two isoforms (GSK3α and GSK3β) have been identified [14]. As a serine/threonine protein kinase, GSK3 regulates a variety of biological processes by phosphorylating a large number of targets. For example, GSK-3 regulates transcription by directly phosphorylating various transcription factors.

As a constitutively active kinase, GSK-3β is inactivated by different signaling pathways. It is also regulated by protein complex formation, particularly in the cytosolic Wnt signaling pathway, where it associates with a large protein complex and phosphorylates *β*-catenin to promote its degradation [14].

Similar to NEMO-deficient animals, mice lacking GSK-3β die during development due to multifocal haemorrhagic degeneration of the liver [15]. These data demonstrate that a loss of GSK-3β results in defective NF-κB signaling in response to TNFα stimulation. During our investigations into the signaling interaction between GSK-3β and NEMO, we were surprised to find that mutations of the GSK-3β phosphorylation sites in NEMO led to a strong reduction in NEMO protein expression and a consequent reduction in NF-κB activation. We therefore hypothesized that NEMO represents a critical link between GSK-3β and NF-κB transcriptional activity.

## 2. Results

### 2.1. IKKγ/NEMO Localization into So-Called “Speckles”

**Figure 1:**
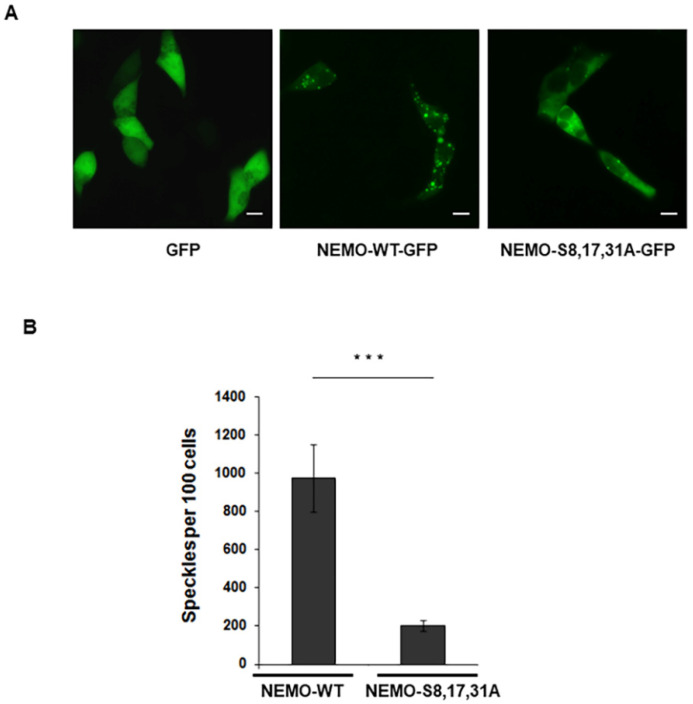
**IKKγ/NEMO localization into so-called “speckles”.** (**A**) Representative microscopic images of HEK293 cells 24 h post-transfection with wild-type NEMO-GFP or mutant S8,17,31A-NEMO-GFP. Scale bar, 10 μm. (**B**) Speckles were counted in at least 100 cells ***, *p* < 0.001.

In a previous report, we showed that GSK-3β participates in IKKγ/NEMO regulation by phosphorylating several serine residues in the N-terminal domain of NEMO. First, we constructed wild-type and mutated constructs of NEMO/IKKγ tagged to GFP and visualized their presentation in living cells. When expressed in HEK293 cells, wild-type GFP-NEMO appeared as multiple cytoplasmic aggregates (Figure 1A) [16]. The mutation of NEMO at Ser8, Ser17 and Ser31 resulted in a dramatic alteration of fluorescence from punctate aggregate to uniform (Figure 1A,B). Furthermore, an increase in punctate fluorescence was observed when cells expressing the GFP-tagged NEMO were co-transfected with wild-type GSK-3β (Supplementary Figure S1), demonstrating that GSK-3β indeed increases formation of NEMO aggregates.

### 2.2. GSK-3-Mediated Phosphorylation Induces the Localization of NEMO into Multivesicular Bodies

**Figure 2:**
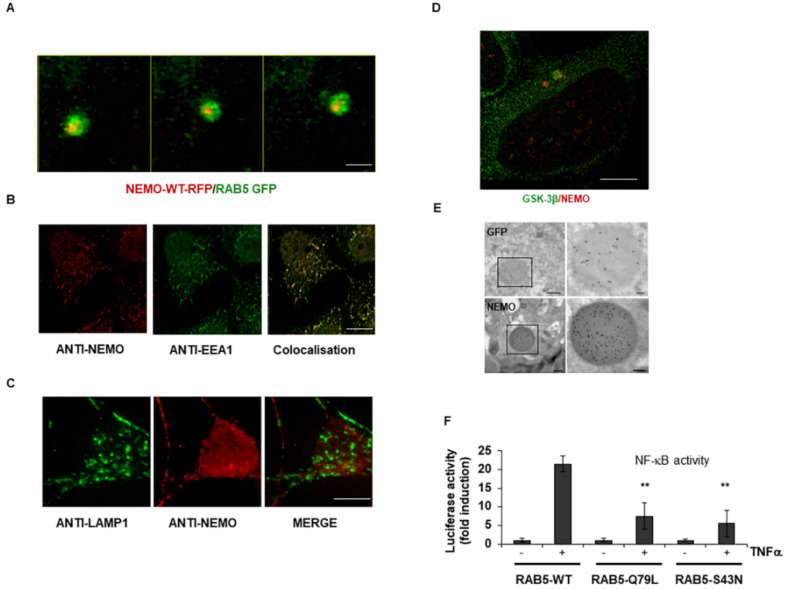
**GSK-3-mediated phosphorylation induces the localization of NEMO into multivesicular bodies.** (**A**) Distribution of NEMO and Rab5 within HEK293 cells after double-transfection with fusion constructs tagged with either GFP (Rab5) or RFP (NEMO). Scale bar, 1 μm. (**B**) Immunofluorescence staining of HEK293 cells with antibodies against EE1 (green) and NEMO (red). Scale bar, 10 μm. (**C**) Immunofluorescence staining of HEK293 cells with antibodies against NEMO (red) and lysosome (green). Scale bar, 5 μm. (**D**) Distribution of endogenous NEMO (red) and GSK-3β (green) visualized by immunostaining of HEK293 cells and subsequent STED imaging. Scale bar, 5 μm. (**E**) Immunoelectron microscopy with the anti-NEMO antibody as well as the anti-GFP antibody clearly labelled round, darkly contrasted areas, often with brighter spots inside the cytoplasm of the transfected cells (only 4% formaldehyde-fixed, not in glutaraldehyde-fixed probes). Scale bar, 0.5 μm and ROI 0.1 μm. (**F**) HEK293 cells were transfected with expression vectors carrying wild-type and Rab5 mutants. After treatment with TNFα (10 ng/mL) for 12 h, NF-κB-dependent gene expression was quantified by measuring luciferase activity. Fold induction is the ratio of stimulated to unstimulated cells (**, *p* < 0.01).

Previous work using TEM and immunofluorescence microscopy showed that GSK-3β within the Wnt pathway localized into multivesicular bodies (MVBs) [17]. Therefore, we first asked whether NEMO and GSK-3β also localized to MVBs. Since the fusion of endosomes leads to MVB formation [18], we performed colocalization studies of NEMO with intracellular organelles. Using the endosome marker Rab5, we observed the localization of NEMO into endosomes (Figure 2A and Video S1). In addition, similar results were observed using EE1 as an endosomal marker (Figure 2B). In contrast, NEMO and Lamp1 did not colocalize, and NEMO was not directed to lysosomes (Figure 2C and Video S2). Using stimulated emission depletion (STED) microscopy, we observed the punctuate expression of endogenous NEMO and the colocalization of NEMO and GSK-3β within the cytoplasm of HEK293 cells (Figure 2D). We further analysed NEMO expression by TEM using immunogold-labelled antibodies, which showed the localization of NEMO into MVBs (Figure 2E). The importance of endosomes in the transcriptional activity of NF-κB signaling was further confirmed by overexpressing either wild-type or mutated Rab5 constructs (Figure 2F).

### 2.3. Selective Inhibitor of V-ATPase Bafilomycin Blocks NF-κB Activity

**Figure 3:**
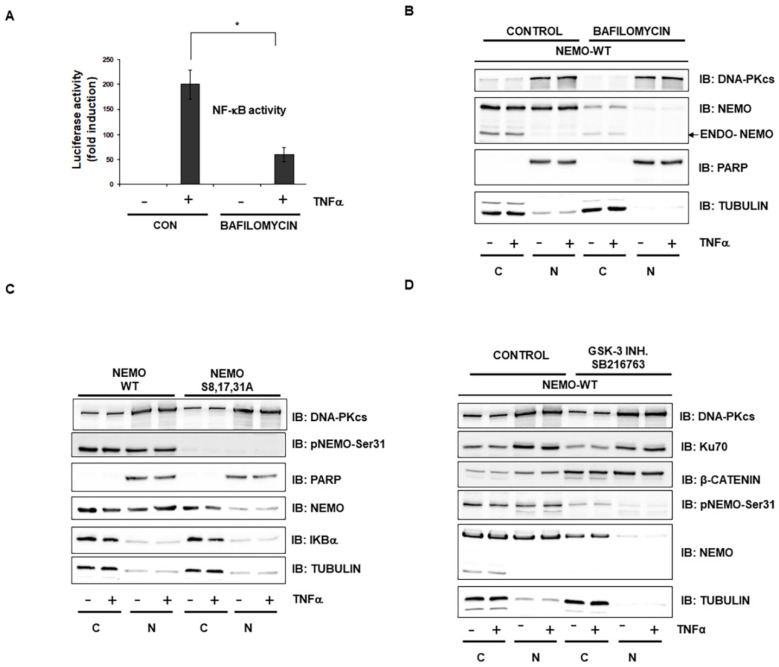
**The selective inhibitor of V-ATPase bafilomycin A blocks NF-κB activity.** (**A**) HEK293 cells were incubated for 24 h with TNFα (1 ng/mL), bafilomycin A (1 µM) and combinations of the two. NF-κB-dependent gene expression was quantified by measuring luciferase activity. Data are expressed as fold changes in luciferase activity compared to the luciferase activity measured in untreated cells. Error bars represent the standard deviation of three experiments (four measurements) *, *p* < 0.05; (**B**) HEK293 cells were transfected with wild-type NEMO. After stimulation with the MVB inhibitor bafilomycin A for 12 h (1 µM), the cells were stimulated with TNFα (10 ng/mL) for 1 h and fractionated; nuclear (N) and cytoplasmic (C) proteins were analyzed by immunoblotting with the indicated antibodies. PARP and tubulin were used as nuclear and cytoplasmic markers, respectively. (**C**) HEK293 cells were transfected with either wild-type NEMO or mutant NEMO. After TNFα treatment (10 ng/mL) for 1 h, the cells were fractionated, and nuclear (N) and cytoplasmic (C) proteins were analyzed by immunoblotting with the indicated antibodies. (**D**) HEK293 cells were transfected with wild-type NEMO. After incubation with the GSK-3 inhibitor SB216763 (1 µM) for 12 h, the cells were stimulated with TNFα (10 ng/mL) for 1 h and fractionated; nuclear (N) and cytoplasmic (C) proteins were analyzed by immunoblotting with the indicated antibodies.

To test the importance of MVBs for NF-κB activity, we treated HEK293 cells with the pharmacologic V-ATPase inhibitor bafilomycin [19] and observed reduced activation of NF-κB as determined by a reporter assay (Figure 3A). As MVBs are involved in the translocation of many viruses from the cytoplasm to the nucleus [20], we also fractionated HEK293 cells after their treatment with bafilomycin and observed the strongly reduced expression of NEMO mainly in the nucleus (Figure 3B). These results observed with bafilomycin were confirmed using NEMO mutant (Figure 3C) and a specific GSK-3 inhibitor (Figure 3D).

### 2.4. Interaction of Vps4A with IKKγ/NEMO

**Figure 4:**
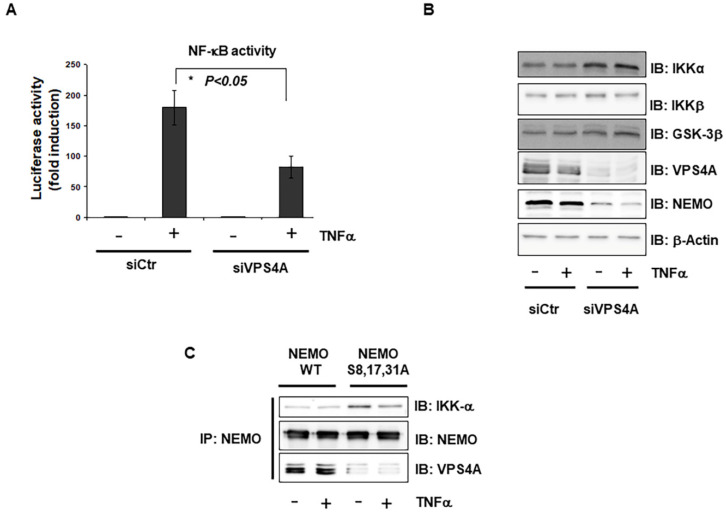
**Interaction between Vps4A and IKKγ/NEMO.** (**A**) Cells were transfected with either GL3 control siRNA or siRNA targeting Vps4A. Where indicated, the cells were treated with TNF-α (1 ng/mL) for 12 h. NF-κB-dependent gene expression was quantified by measuring luciferase activity. Data are expressed as fold changes in luciferase activity compared to the luciferase activity measured in untreated cells. Error bars represent the SD of three experiments (four measurements) *, *p* < 0.05; (**B**) Cells were transfected with either GL3 control siRNA or siRNA targeting Vps4A. Where indicated, the cells were treated with TNF-α (1 ng/mL) for 12 h. The lysates were immunoblotted (IB) with the indicated antibodies. β-Actin was used as a loading control. (**C**) HEK293 cells were transfected with wild-type NEMO or S8,17,31A mutant NEMO. Lysates from TNFα-stimulated (10 ng/mL) cells were immunoprecipitated (IP) with anti-GFP, followed by immunoblotting (IB) with anti-NEMO, anti-IKKα, and anti-Vps4A.

The sorting of endosomal membrane proteins into the MVB pathway is executed by the class E Vps protein complexes ESCRT-I, -II, and -III and the AAA-type ATPase Vps4 [21]. Therefore, to confirm the results obtained with the MVB inhibitor, we first used siRNAs to target two subunits of V-ATPase that function in the biogenesis of MVBs (Vps4A and Vps4B). Interestingly, we observed a strong reduction in NF-κB activation only with Vps4A knockdown (Figure 4A). Furthermore, the silencing of Vps4A led to a strong reduction in NEMO protein expression, while a slight increase in other components of the NF-κB pathway, such as IKKs, IKBα and GSK-3β, was observed (Figure 4B). To test for the direct interaction of NEMO with Vps4A, the co-immunoprecipitation of wild-type NEMO and triple-mutated NEMO-S8,17,31A demonstrated that Vps4A and NEMO interact directly and that this interaction requires the phosphorylation of NEMO (Figure 4C).

### 2.5. Vps4A Is a Key Enzyme That Modulates NEMO Expression and Function

**Figure 5:**
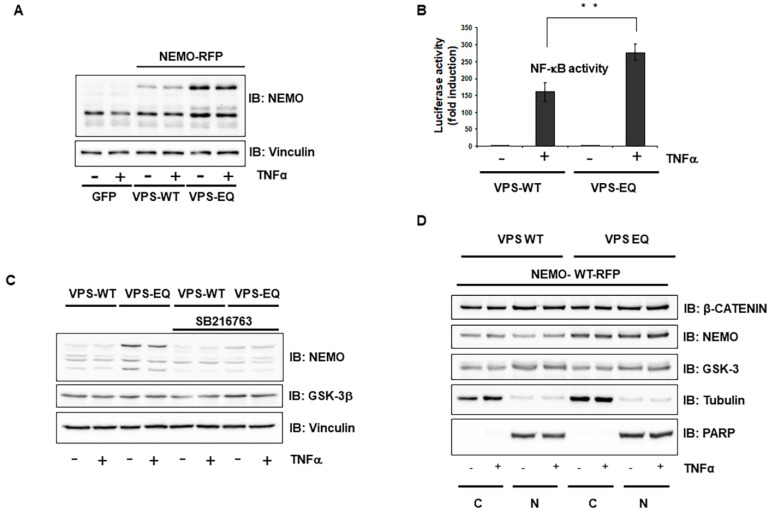
**Vps4A is a key enzyme that modulates NEMO expression and function.** (**A**) HEK293 cells were co-transfected with wild-type NEMO and either wild-type Vps4 or mutant Vps4A-E233Q. Where indicated, the cells were treated with TNF-α (10 ng/mL) for 1 h. The lysates were immunoblotted (IB) with the indicated antibodies. (**B**) HEK293 cells were co-transfected with wild-type NEMO and either wild-type Vps4 or mutant Vps4A-E233Q. After treatment with the GSK-3 inhibitor SB216763 (1 µM) for 12 h, cells were stimulated with TNFα (10 ng/mL) for 1 h. Cell lysates were assayed for the expression of NEMO. (**C**) HEK293 cells were transfected with wild-type or mutant Vps4A-E233Q. After stimulation with TNFα (1 ng/mL), NF-κB-dependent gene expression was quantified by measuring luciferase activity. Data are expressed as fold changes in luciferase activity compared to the luciferase activity measured in untreated cells. Error bars represent the SD of three experiments. ** *p* < 0.01; (**D**) HEK293 cells were co-transfected with wild-type NEMO and either wild-type Vps4A or mutant Vps4A-E233Q. The cells were stimulated with TNFα (10 ng/mL) for 1 h and fractionated; nuclear (N) and cytoplasmic (C) proteins were analyzed by immunoblotting with the indicated antibodies. PARP and tubulin were used as nuclear and cytoplasmic markers, respectively.

In addition, we used a mutated form of Vps4A (Vps4A-E233Q), previously shown to be devoid of ATPase activity, to trigger the formation of enlarged endosomal structures called class E compartments [20,22]. The transfection of cells with GFP-Vps4A-E233Q resulted in the formation of large vacuoles (Supplementary Video S4 and Supplementary Figure S1C), which were readily identified against the background fluorescence of cytosolic GFP-Vps4-WTA (Supplementary Video S3 and Supplementary Figure S1C). The increased amount of vacuolation led to a strong augmentation in NEMO expression in comparison to its expression in cells transfected with wild-type GFP-Vps4-WT (Figure 5A). This upregulation of NEMO in the Vps4A-E233Q mutant was blocked by a specific GSK-3 inhibitor (Figure 5B). Furthermore, the overexpression of mutant Vps4A-E233Q led to the increased NF-κB activity (Figure 5C) and augmentation of NEMO even in the nucleus, as shown after the fractionation of co-transfected HEK293 cells, confirming the importance of Vps4A in NEMO regulation (Figure 5D).

Our results demonstrated that NEMO requires localization into multivesicular bodies and V-ATPase activity for stabilization and subsequent NF-κB activation.

## 3. Discussion

In our previous report, we identified NEMO as a new GSK-3β substrate that is phosphorylated at several serine residues within the N-terminal domain of NEMO. In this report, we provide evidence that NEMO localizes in multivesicular bodies, and this has enormous importance for its downstream signaling towards NF-κB. NEMO expression was previously visualized into so-called “speckles” [16], but the consequence of this localization has not been investigated. Here, we report that bafilomycin, a specific inhibitor of the vacuolar proton ATPase, does not change GSK-3β protein expression but leads to a dramatic downregulation of NEMO thereby inhibiting NF-κB signal transduction. Using siRNAs targeting Vps4 proteins, we identified the AAA-ATPase Vps4A, which is involved in recycling the ESCRT machinery by facilitating its dissociation from endosomal membranes [23], as necessary for NEMO stability and NF-κB activity. Using a mutated form of Vsp4A, Vps4AE223Q, we observed the strong upregulation of NEMO, and a consequent increase in NF-κB activity. Our results were in concordance with those observed in other studies using the Vps4A-E223Q mutant in which discrete punctuate structures disappeared, and enlarged endosomal structures were observed by the overexpression of Vps4A-E223Q [21].

MVBs were initially identified as part of the protein degradative pathway [24]. In our study, MVB formation did not appear to simply be a mechanism for degradation since we did not see any colocalization of NEMO with lysosomes or with autophagy marker proteins such as LC3 (not shown). As multivesicular bodies have been demonstrated to play an important role in cellular processes such as proliferation, inflammation and apoptosis, our study opens new aspects in the study of MVB formation regarding NF-κB function.

Furthermore, our results suggest that MVBs are necessary for NEMO stability and that the ESCRT machinery is required for efficient NEMO degradation. The relocalisation of GSK-3β from the cytosol into prominent cytoplasmic puncta after Wnt treatment and the sequestration of GSK-3β into MVBs, resulting in the modulation of the β-catenin pathway, has been shown previously [17]. In our study, we observed the colocalization of GSK-3β and NEMO in quiescent cells, and no reduced expression of GSK-3β was observed after using either an MVB inhibitor or siRNA against Vps4A. Interestingly, after the co-transfection of NEMO and GSK-3β, an increase in GSK-3β accumulation into the MVBs was observed over time. This result indicated that the phosphorylation of NEMO by GSK-3β led to the simultaneous recruitment of NEMO and GSK-3β into MVBs. The function of GSK-3β in different pathways often depends on its substrate, and we postulated that the function and effects of GSK-3β on NF-κB correlate strongly with NEMO function.

It remains unclear how NEMO can be directed into MVBs. The recruitment of NEMO to the T-cell Receptor (TCR) was previously reported, and this signalosome is necessary for TCR signaling after TNFα stimulation [25]. This type of recruitment may be a mechanism by which NEMO is directed to MVBs. This study provides the first report of a molecular mechanism by which an inhibitor of MVB formation and GSK-3 blocked the expression of NEMO and, in this way, blocked NF-κB activity.

It is well known that endogenous NEMO is localized predominantly in the cytoplasm. However, the post-translational modification of NEMO, including its SUMOylation, phosphorylation, and ubiquitination, also occurs in the nucleus following exposure to DNA damage [9,10,26]. The mechanism underlying the translocation of NEMO into the nucleus is not clear, since NEMO lacks a canonical nuclear localization signal. GSK-3β plays a role in the shuttling of many proteins from the cytoplasm to the nucleus and vice versa [27]. For example, androgen receptor phosphorylation by GSK-3 results in their stabilization and translocation from the cytoplasm into the nucleus. In a recent study [28], it was shown that the phosphorylation of an S/T-P-S/T domain acted as a general nuclear translocation signal, as is the case for Ser-8,17/Ser31 in NEMO. Therefore, one could speculate that this site is responsible for the nuclear localization of NEMO. Such a nuclear translocation signal is found in many other substrates of GSK-3β, such as oestrogen [29] or androgen receptors [30]. However, further studies are needed to understand the precise mechanism by which NEMO nuclear translocation is controlled.

Collectively, our data suggest that the function of NEMO is positively regulated by its phosphorylation through GSK-3β and that this novel regulation mechanism sheds new light on how GSK-3β modulates NF-κB activation.

## 4. Material & Methods

### 4.1. Cell Culture and Transfection

HEK293 cells (DSMZ, Braunschweig, Germany) were grown in Dulbecco’s modified Eagle’s medium (4.5 g/L glucose; Invitrogen) containing 1% foetal bovine serum (FCS) and 1 mM pyruvate (22) for 48 h before stimulation with TNFα. HEK293 cells were transfected by the calcium phosphate method. Briefly, HEK293 cells were grown to ca. 60–70% confluence and mixed with a calcium phosphate-DNA precipitate consisting of plasmid DNA, 2.5 M CaCl_2_ and 2× HBS buffer. The transfection efficiency of all cell lines was >90%, as proven by a green fluorescent protein (GFP) control vector.

### 4.2. Reagents and Antibodies

The following antibodies and reagents were used: anti-GSK-3β (#9832) and an NF-κB Pathway Sampler Kit (#9936) (New England Biolabs, Frankfurt/Main, Germany), phosphor-specific anti-pSer-31-NEMO and anti-β-actin (Abcam, Cambridge, UK), anti-Vps4A (#SAB4200025) and mouse anti-FLAG M2 (Sigma-Aldrich, Taufkirchen, Germany), and anti-tubulin and nonimmune IgGs (Invitrogen, Darmstadt, Germany). Recombinant human TNFα was purchased from Miltenyi (Bergisch Gladbach, Germany), bafilomycin A (#B1793) was purchased from Sigma-Aldrich, and the GSK-3β inhibitor SB216730 was purchased from Calbiochem (San Diego, CA, USA).

### 4.3. Luciferase Assay

HEK293 cells stably transfected with 5×NF-κB-RE were washed with phosphate-buffered saline (Mg^2+^- and Ca^2+^-free) and lysed in 150 µL/well luciferase cell culture lysis reagent (Promega, Mannheim, Germany). Luciferase assays were performed using the luciferase assay system from Promega according to the manufacturer’s instructions and quantified with a luminometer (LB9506, Berthold, Bad Wildbad, Germany).

### 4.4. Immunoprecipitation

The immunoprecipitation of GFP-tagged proteins was performed using a GFP Isolation Kit (#130-091-125) according to the manufacturer’s instructions (Miltenyi, Bergisch Gladbach, Germany).

### 4.5. Gene Silencing with Small Interfering RNAs

The transfection of HEK293 cells with siRNA was performed using Lipofectamine 2000 (Invitrogen, Darmstadt, Germany) according to the manufacturer’s instructions. siRNA oligonucleotides with 3′-TT overhangs were purchased from MWG-Biotech AG (Ebersberg, Germany). The following siRNA sequences were used: siVps4A-1, 5′-CCG AGA AGC UGA AGG AUU A dTdT-3; siVps4A-2, 5′-GCA AAG AGA AAC ACG GCA A dTdT-3; siVps4B-1, 5′-CCA AAG AAG CAC UGA AAG A dTdT-3; and siVps4B-2, 5′-GGA UGU CCC UGG AGA UAA A dTdT-3. The siRNA concentration was 20 nM during transfection. For a control, we used GL-3-targeted siRNA. The transfection efficiency was >70%.

### 4.6. Plasmids

The plasmids used to clone GSK-3β have been described [29]. The full-length cDNA of human NEMO was cloned into pTagGFP-N or pTagRFP-N (Evrogen). Site-directed mutagenesis of NEMO (serine to alanine) was performed using the QuikChange site-directed mutagenesis kit from Stratagene (La Jolla, CA, USA). The mutations were verified by DNA sequence analysis. In addition, we used the BacMan system for the transfection of GFP-tagged Lamp1 (#C10596) or Rab5 (#C10586) according to the manufacturer’s instructions (Invitrogen, Darmstadt, Germany).

### 4.7. Preparation of Nuclear Extracts

The preparation of nuclear extracts has been described previously [29].

### 4.8. Staining of Cell Cultures for STED Microscopy

The HEK293 cells were fixed with 4% paraformaldehyde for 10 min at room temperature (RT) followed by 3 washes with PBS. Incubation with a primary antibody was performed overnight at 4 °C. For secondary antibody labelling, after removal of the primary antibodies, the cells were washed 5 times with PBS and then incubated with fluorescence-labelled secondary antibodies in blocking buffer. The following secondary antibodies were used: Atto 647 N anti-rabbit diluted 1:200 and Chromeo 494 anti-mouse diluted 1:50 (Active Motif, La Hulpe, Belgium). Incubation was carried out for 1 h at RT, followed by another 5 washes. Finally, the cells were embedded in Mowiol at pH 8.4.

### 4.9. Image Acquisition of Stained Cell Cultures by STED Microscopy

For confocal and STED imaging of the Chromeo 494 and Atto 647 N dyes with emission maxima of approximately 600 nm (channel 1) and 680 nm (channel 2), respectively, we used a 2-channel TiSphr.-pulsed STED microscope (Leica Microsystems, Germany) equipped with a 100× Oil Plan Apo NA 1.4 STED objective. Confocal as well as STED images were recorded by scanning the focused beam with a galvo mirror at 1000 Hz with 64-line average and a pixel size of 25 nm across the specimen. In STED mode, a pixel size of 25 nm was achieved with the 100× Oil NA 1.4 objective at an image format of 1024 pixels using a zoom factor of 6.

Typically, a series of images consisted of 4 channels: channel 1: Atto 647 N (LSM mode), channel 2: Chromeo 494 (LSM mode), channel 3: Atto 647 N (STED mode), and channel 4: Chromeo 494 (STED mode).

### 4.10. Image Processing

To improve image quality, the raw confocal and STED images were deconvolved using Autoquant deconvolution software (Media Cybernetics Inc., Rockville, MD, USA) with a theoretical PSF. Subsequently, the images were processed using ImageJ (National Institutes of Health, Bethesda, MD, USA) by merging channels and converted into 8-bit RGB images. The contrast and brightness levels of individual channels were finally adapted by Photoshop CS 5 (Adobe. System Inc., San Jose, CA, USA),

### 4.11. Immunoelectron Microscopy

HEK293 cells transfected with NEMO-WT GFP or untransfected HEK293 cells (as controls) were fixed with 4% formaldehyde (FA) for 4 h or with 2% FA and 0.2% glutaraldehyde in 0.1 M phosphate buffer (PB) for 1 h, washed carefully with phosphate-buffered saline (PBS), covered with 1% gelatin in PBS, scraped from the dishes and transferred to Eppendorf tubes. After the cells were spun down (10 min, 210× *g*), the pellet was suspended in 12% gelatin in PBS at 37 °C for 5 min, centrifuged and placed on ice for 30 min. The solidified cell pellet was cut into cubes of approximately 1 mm^3^ and incubated in 2.3 M sucrose in PBS overnight for freeze protection. The next day, the specimen blocks were placed on specimen holders and frozen by immersing them in liquid nitrogen. Thin cryosections were prepared with a UCT (Leica) and labelled according to an immunogold technique used on thawed cryosections originally introduced by Tokuyasu [31] and modified by Griffiths et al. [32] and Slot and Geuze [33].

### 4.12. Widefield FLIM Setup

The widefield FLIM setups used for our FRET experiments have been described previously [34].

### 4.13. Statistical Analysis

Data are given as mean ± SEM. Comparisons across multiple groups were performed by ordinary one-way ANOVA. Experiments with two groups were analyzed with Student’s *t*-test. All experiments were independently repeated at least three times.

List of abbreviations: ESCRT, endosomal sorting complexes required for transport; FLIM, fluorescence lifetime imaging microscopy; FRET, fluorescence resonance energy transfer; GSK, glycogen synthase kinase; MVBs, multivesicular bodies; PBS, phosphate-buffered saline; PSF, point spread function; RE, response element; RT, room temperature; STED, stimulated emission depletion; TCR, T cell receptor; TEM, transmission electron microscopy; V-ATPase, vacuolar proton ATPase.

## Data Availability

Not applicable.

## Supplementary Materials

The following supporting information can be downloaded at: www.mdpi.com/article/10.3390/ijms23126778/s1.
