# Supplementary material for: IKKγ/NEMO Localization into Multivesicular Bodies
Source: Int J Mol Sci. 2022 Jun 17;23(12):6778. doi: 10.3390/ijms23126778 (PMC9223704; doi:10.3390/ijms23126778)
Supplement: Supplementary file 1 [file ijms-23-06778-s001.zip › ijms-1752132-supplementary-final/Suppl. Information Videos Suppl. Figures/Supplementary Figures.docx]

**
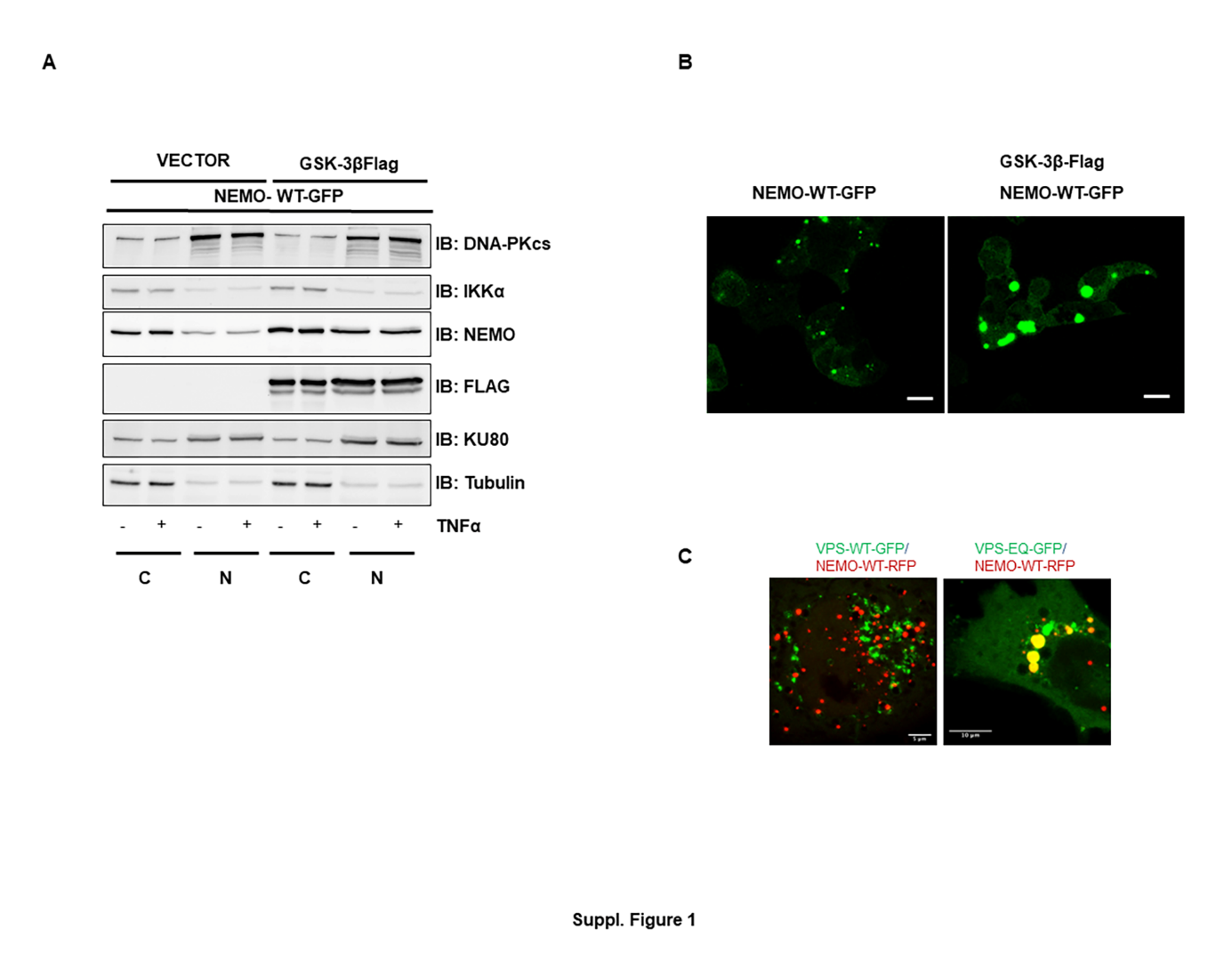
Supplementary Figure S1. GSK-3β increases NEMO expression** (**A**) HEK293 cells were co-transfected with wild-type NEMO and either wild-type GSK-3β-Flag or empty vector. After stimulation with TNFα (10 ng/ml) for 1 h and fractionated; nuclear (N) and cytoplasmic (C) proteins were analysed by immunoblotting with the indicated antibodies. (**B**) Representative microscopic images of HEK293 cells 24 h post-transfection with wild-type NEMO-GFP and either wild-type GSK-3β-Flag or empty vector. Scale bar. 10 μm. (**C**) Representative microscopic images of HEK293 cells 24 h post-transfection with wild-type NEMO-RFP and either and either wild-type or mutant Vps4A-E233Q.

**
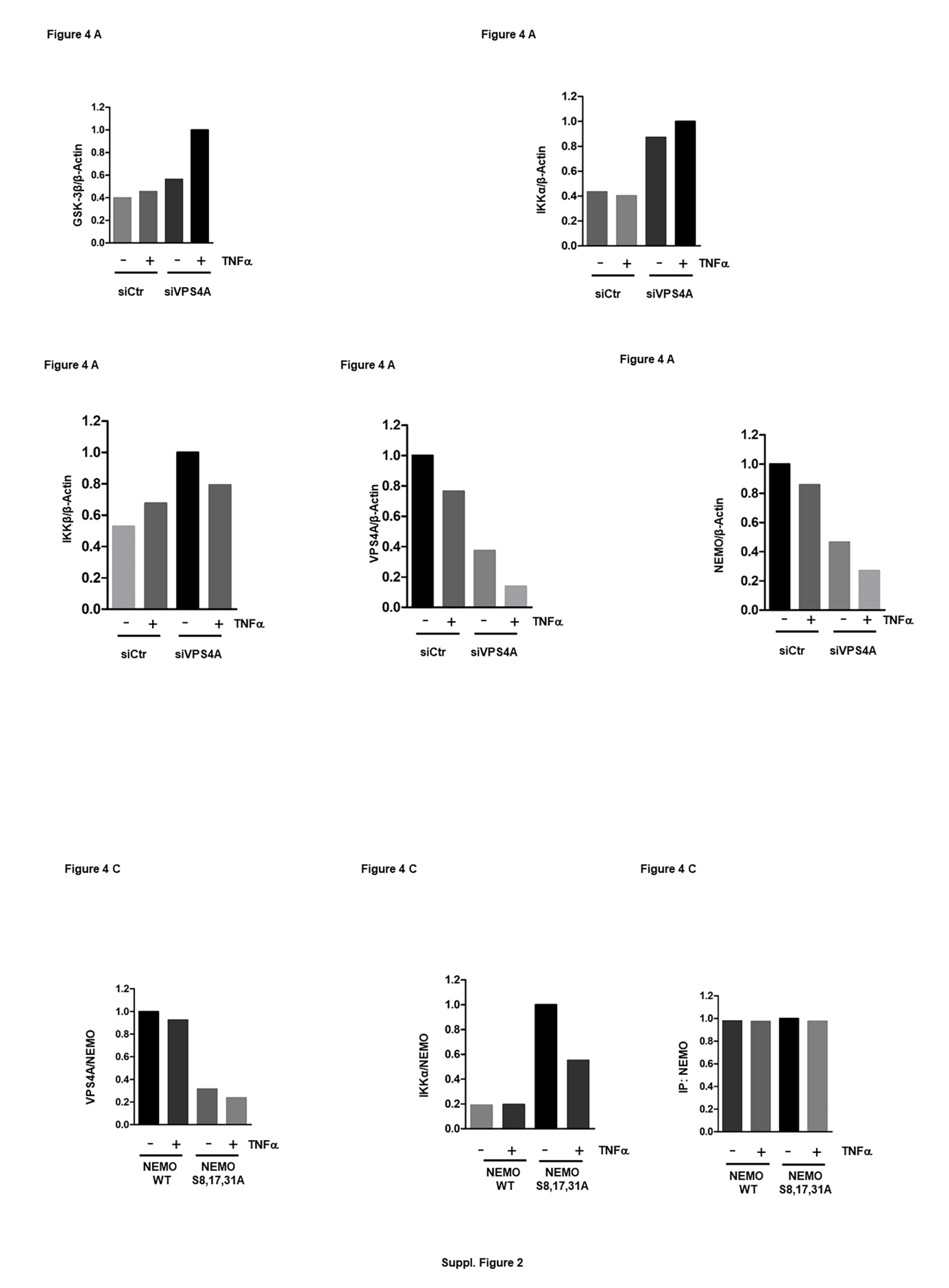
**

**Supplementary Figure S2. Densitometric quantifications of Western Blots Figure 4.**

**
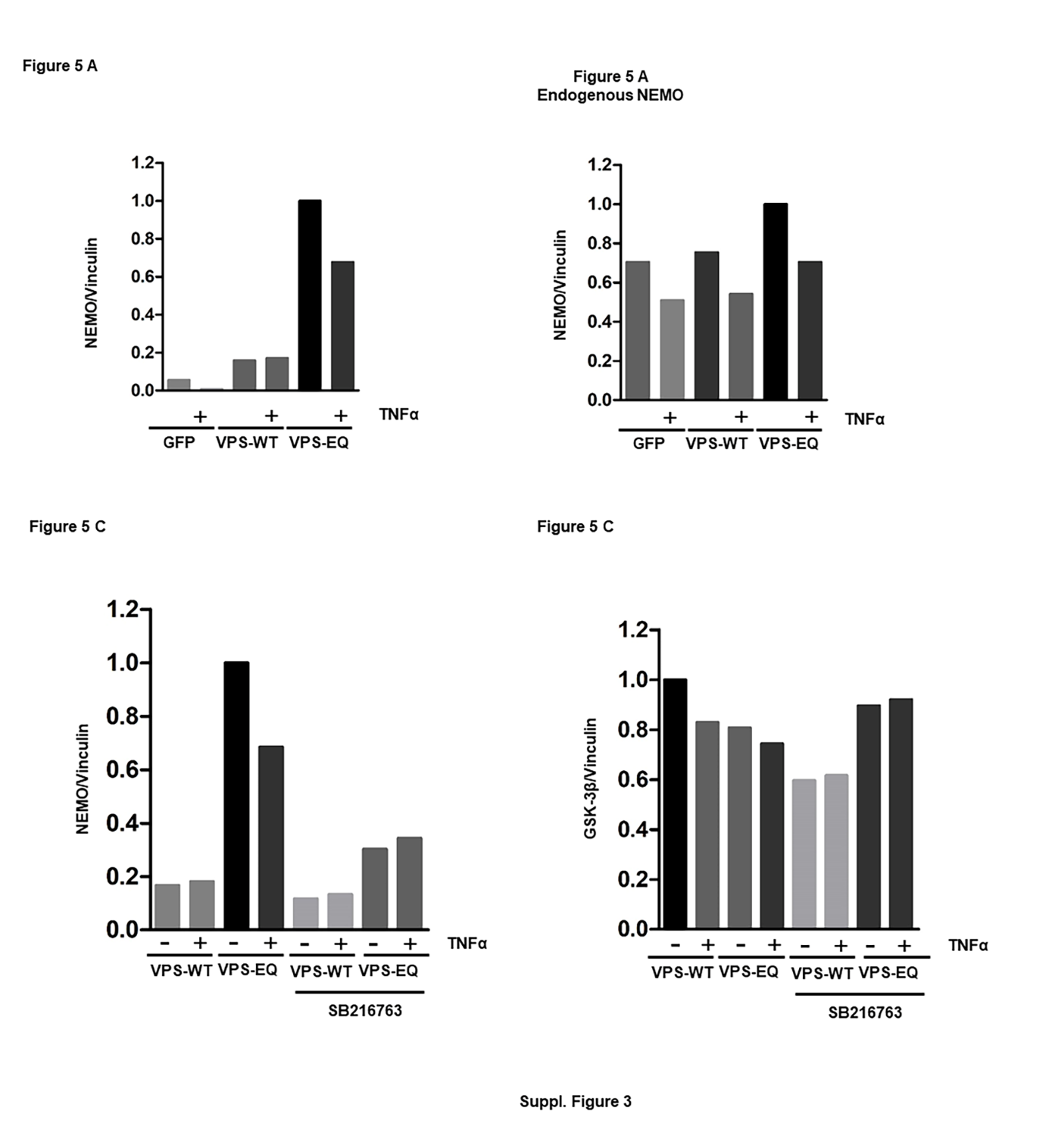
**

**Supplementary Figure S3. Densitometric quantifications of Western Blots Figure 5.**

**Supplementary Video S1.** **Time lapse imaging of overexpressed NEMO-Rab5.** Distribution of NEMO and Rab5 within HEK293 cells after double-transfection with fusion constructs tagged with either GFP (Rab5) or RFP (NEMO). The time lapse series shows labeling of Rab5 positive vesicular structures that were attached by NEMO or contain intraluminar NEMO.

**Supplementary Video S2.** **Time lapse imaging of overexpressed NEMO-lysosome marker.** Life imaging of co-transfected NEMO (RFP) and Lysosome (Lamp1) (GFP) did not show any coexpression. On the contrary both markers were localized in different vesicular structures moving throughout the cytoplasm.

**Supplementary Video S3. Distribution of NEMO and Vps4A-WT.** Life imaging of HEK293 cells after double-transfection with fusion constructs tagged with either GFP (Vps4A) or RFP (NEMO). The time lapse series shows labeling of Vps4A positive vesicular structures that were attached by NEMO.

**Supplementary Video S4. Distribution of NEMO and Vps4A-E233Q**. Life imaging of HEK293 cells after double-transfection with fusion constructs tagged with either GFP (Vps4A-E233Q) or RFP (NEMO). The time lapse series shows large Vps4A positive vesicular structures that contain intraluminar NEMO.
